# Primary care for people with an intellectual disability — what is prescribed? An analysis of medication recommendations from the BEACH dataset

**DOI:** 10.3399/bjgpopen18X101541

**Published:** 2018-05-30

**Authors:** Carmela Salomon, Helena Britt, Allan Pollack, Julian Trollor

**Affiliations:** 1 Postdoctoral Project Officer, Department of Developmental Disability Neuropsychiatry, School of Psychiatry, UNSW, Sydney, Australia; 2 Professor of Primary Care Research and Director, Family Medicine Research Centre, School of Public Health, Faculty of Medicine, University of Sydney, Sydney, Australia; 3 Research Analyst, Family Medicine Research Centre, School of Public Health, Faculty of Medicine, University of Sydney, Sydney, Australia; 4 Chair of Intellectual Disability Mental Health and Head of Department of Developmental Disability Neuropsychiatry, School of Psychiatry, UNSW, Sydney, Australia

**Keywords:** intellectual disability, general practice, prescriptions, psychotropic drugs, narcotics, antihypertensive agents

## Abstract

**Background:**

People with intellectual disability (ID) experience a range of health disparities. Little is known about differential primary care prescribing patterns for people with and without ID.

**Aim:**

To compare medications recommended by GPs at encounters where ID is recorded versus other encounters.

**Design & setting:**

Analysis of national Australian GP medication data from the Bettering the Evaluation and Care of Health (BEACH) programme, January 2003–December 2012 inclusive.

**Method:**

Medication recommendations made at encounters where an ID-defining problem was recorded as a reason for encounter (RFE) and/or as a problem managed, were allocated to the ‘ID group’ (*n* = 563). These encounters were compared with all other encounters (the ‘non-ID group’, *n* = 1 004 095) during the study period. Following age–sex standardisation of ID group encounters, significant differences were determined by non-overlapping 95% confidence intervals (CIs).

**Results:**

Antipsychotics and anticonvulsants were recommended more frequently at ID group encounters than at non-ID group encounters. Antidepressant and anxiolytic recommendation rates did not differ between groups. Narcotic analgesic and antihypertensive recommendations were significantly lower at ID group encounters.

**Conclusion:**

Higher rates of epilepsy and mental illness, and off-label use of some antipsychotics and anticonvulsants for behaviour management in people with ID, may have contributed to medication recommendations observed in this analysis. Lower narcotic analgesic recommendations at ID group encounters may relate to complex presentations and the nature of problems managed, while lower antihypertensive recommendations may indicate some potential omission of routine blood pressure measurement.

## How this fits in

This is the first study to directly compare medicines prescribed, supplied, or recommended at GP encounters for people identified at encounter as having ID and those who were not, using continuous nationally-representative general practice data. Between-group differences in psychoactive medication recommendation rates may relate to epidemiological factors, diagnostic challenges, and off-label recommendations in the ID group. Complexities related to pain assessment in this population may be reflected in lower than expected rates of narcotic analgesic recommendations, while lower antihypertensive recommendations suggest some core physical healthcare needs of people with ID may still be overlooked in general practice.

## Introduction

Globally, the past few decades have delivered meaningful improvement in health and wellbeing for people with an ID.^1^ Yet people with ID continue to experience premature mortality^2^ and major access barriers to quality primary health care,^3^ and do not always receive the quality health care they are entitled to under Article 25 of the United Nations Convention on the Rights of People with Disabilities.^4^ Research in general practice indicates that, on average, people with ID have 2.5 times the number of health problems compared to people without ID.^5^ Both psychiatric disorders and physical health problems are over-represented in people with ID.^6^ However, many health problems are undetected and therefore not actively managed in this population.^3^

Despite a central role in the healthcare management of people with an ID,^3^ many GPs lack specific education and training in ID health,^7^ and report low confidence in their ability to provide health care for this population.^3^ A number of administrative and practical constraints may impede delivery of preventative health care.^8^ For example, GPs have identified difficulties communicating effectively both with patients with ID and their carers.^3^ Examining how GPs manage health problems in patients with ID, including what medications they prescribe, may generate knowledge about gaps in GP practice or skills.

The higher prevalence of physical and psychiatric comorbidities among people with ID^6^ would suggest that general practice visitation and prescribing rates should be higher for people with ID than for the general population. In line with this supposition, Straetmans and colleagues^9^ used data from the Second Dutch National Survey of General Practice to determine that people with ID visited GPs more frequently than matched controls, and received more repeat prescriptions. However, somewhat surprisingly, per consultation they reported that people with ID were less likely to receive a prescription. A recently published analysis from the Australian BEACH data set also reported the surprising finding that fewer overall medication recommendations were made at ID general practice encounters.^8^ Neither Straetmans^9^ nor Weise^8^ reported prescription rates by medication class or group. There is therefore a need to clarify which, if any, types of medications may be under- or overprescribed to people with ID in general practice. Such an analysis may help to inform future general practice education.

The BEACH programme provides a mechanism to explore and compare nationally representative primary care medication data for face-to-face and phone encounters between GPs and patients at which ID is recorded ('ID group’) versus other (‘non-ID group’) encounters. Through analysis of the BEACH dataset, this study addresses the research question: do the types of medication recommended at ID group encounters differ from those at non-ID group encounters? The authors hypothesised that the types of medications recommended at ID group encounters would differ from those at non-ID group encounters, according to known patterns of medical and psychiatric comorbidities experienced by people with ID.

## Method

### Sample

This analysis uses BEACH data collected from January 2003–December 2012 inclusive. BEACH methods are described in detail elsewhere^10^ but are summarised here for convenience. The BEACH study provides a continuous national picture of general practice activity in Australia. Approximately 1000 ever-changing, randomly sampled GPs take part in each year. Each participating GP documents details of 100 consecutive encounters with consenting, de-identified patients on structured paper-based forms. Carers are able to consent on behalf of patients who lack capacity to self-consent. RFEs and problems managed are secondarily coded in the Australian general practice terminology known as ICPC-2 PLUS,^11,12^ which is classified according to the International Classification of Primary Care (ICPC-2).

Participating GPs are asked to record at least one, and up to four, problems managed at the encounter, with a maximum of four medications recorded per problem, and to indicate whether each of these medications was ‘prescribed’, ‘GP-supplied’, or ‘recommended for over the counter purchase’. For the purposes of this report, these medication pathways are collectively referred to as medications ‘recommended’ by the GP. An in-house BEACH system, the Coding Atlas for Pharmaceutical Substances (CAPS) is used to classify all recorded medications based on anatomical site and therapeutic use. CAPS allows analysis at the group, sub-group, generic (composition of drug), proprietary brand name, product, and form levels. This paper reports on medication recommendations at the CAPS group, sub-group, and generic levels. While medications in the CAPS coding system are mapped to the Anatomical Therapeutic Chemical (ATC) classification, the CAPS system provided greater specificity than the ATC equivalent in certain cases. For example, at the CAPS group level, ‘psychological’ medications can be analysed separately from 'central nervous system (CNS)' medications, whereas the ATC level 1 equivalent ‘nervous system’ category does not provide this specificity. In order to maximise the clinical utility of the data, this study therefore reports medication findings according to the CAPS classification.

Any encounter where ≥1 ID-defining problem was recorded as a patient RFE and/or as a problem managed at encounter was defined as an ‘ID group’ encounter. The ID-defining RFEs and problems were all those classified in ICPC-2 as 'P85 mental retardation', and selected ICPC-2 PLUS codes forming part of ICPC-2 'A90 congenital anomaly not otherwise specified/multiple', namely A90001 (Down syndrome) and A90015 (Fragile X syndrome). All other encounters that occurred during the study timeframe were defined as non-ID group encounters. By implication, all patients at ID group encounters had, or were being assessed for, a diagnosis of ID. Although it is likely that a small proportion of encounters in the non-ID group were with patients with ID, the large size of this group would mean that the inadvertent inclusion of these individuals in the non-ID group would have minimal impact on results. Medication recommendations made at ID group and at non-ID group encounters were compared.

### Statistical analysis

A single-stage cluster design is used in the BEACH programme and all analyses are adjusted for clustering (at the level of participating GP).^10^

The χ^2^ test was used to determine the statistical significance of demographic differences between patients in the ID group and the non-ID group. To allow for meaningful between-group comparisons, data at ID group encounters were weighted to model the results expected if the age–sex distribution of patients at ID group encounters was matched to that at non-ID group encounters ('age–﻿sex standardisation').

The rates of recommendation of medications per 100 encounters were analysed at the CAPS group, sub-group, and generic medication level, and the ID group and non-ID group were compared. Between-group differences were considered significant if there was no overlap in the 95% CIs. This is equivalent to *P*<0.006^13^ for each comparison, which is more conservative than the usual *P*<0.05. The statistical software package SAS (version 9.3) was used.

## Results

### Patient demographics

Of a total of 971 331 BEACH encounters with a specified patient age, 690 (0.07%) were classed as ID encounters. The age and sex distribution of patients differed between the ID group and non-ID group, the ID group having over-representation of patients aged 5–44 years, and of males (Table 1). The over-representation of males at ID group encounters may be partially explained by the higher incidence of ID in men compared to women.^14^ There were 563 medications recommended at ID group encounters and 1 004 095 at non-ID group encounters.

**Table 1.** Patient sex and age distributions in ID and non-ID group encounters

		ID group patients (*n* = 690)		Non-ID group patients (*n* = 970 641)	
		***n *(%)**	**95% CI**	***n *(%)**	**95% CI**
Sex (Missing data: ID, *n* = 9; non-ID, *n* = 6968)	Male	348 (51.1)	47.2 to 55.0	392 719 (40.8)	40.5 to 41.0
	Female	333 (48.9)	45.0 to 52.8	570 954 (59.2)	59.0 to 59.5
Age group, years	<5	26 (3.8)	2.3 to 5.3	61 753 (6.4)	6.3 to 6.5
	5–14	78 (11.3)	8.6 to 14.0	51 987 (5.4)	5.3 to 5.4
	15–24	104 (15.1)	12.0 to 18.1	86 392 (8.9)	8.8 to 9.0
	25–44	223 (32.3)	28.6 to 36.0	226 095 (23.3)	23.1 to 23.5
	45–64	212 (30.7)	26.8 to 34.7	269 729 (27.8)	27.6 to 28.0
	65–74	30 (4.3)	2.7 to 6.0	123 370 (12.7)	12.6 to 12.9
	≥75	17 (2.5)	1.2 to 3.7	151 315 (15.6)	15.3 to 15.8

There are highly significant differences between the ID and non-ID groups:

For sex: *P*< 0.001; Rao-Scott χ^2^ adjusted for GP cluster effect (RS-χ^2^) 28.7; 1 df.

For age-group: *P*<0.001; RS-χ^2^ 186.6; 4 df.

Encounters with missing age data excluded from above table: ID 11/701; non-ID 8058/978 699.

CI = confidence intervals. ID = intellectual disability.

### Medication ‘group’ recommendations (CAPS ‘group’ level)

All results are presented as medications per 100 encounters, with 95% CIs. After age–sex standardisation, patients at ID group encounters were more frequently recommended medications in the ‘psychological’ group (12.6 [95% CI = 8.8 to 16.3]) than those at non-ID group encounters (7.9 [95% CI = 7.8 to 8.1). Patients at ID group encounters were recommended significantly fewer medications in the 'hormones' group (3.5 [95% CI = 2.1 to 4.9] versus 5.7 [95% CI = 5.6 to 5.8]), 'cardiovascular' group (3.0 [95% CI = 1.3 to 4.7] versus 15.3 [95% CI = 15.0 to 15.5]), and 'skin' group of medications (2.2 [95% CI = 1.1 to 3.2] versus 3.7 [95% CI = 3.7 to 3.8]).

### Medication ‘subgroup’ recommendations (CAPS ‘subgroup’ level)

Table 2 indicates medication patterns by comparing the top 10 ranked medication subgroups in the ID and non-ID groups of encounters. Of the top 10 medication CAPS subgroups at ID group encounters, only six were shared with the non-ID group top 10 list. Compared with patients at non-ID group encounters, those at ID group encounters were recommended significantly more medications in the ‘antipsychotic’ CAPS subgroup (6.1 [95% CI = 3.7 to 8.5] versus 0.6 [0.6 to 0.6]) and the ‘anticonvulsant’ CAPS subgroup (6.0 [95% CI = 3.2 to 8.7] versus 0.6 [95% CI = 0.6 to 0.6]). In contrast, recommendation rates at ID group encounters were significantly lower for the ‘other antibiotics’ subgroup (1.7 [95% CI = 0.7 to 2.6] versus 3.0 [95% CI =3.0 to 3.1]) and the *'*antihypertensives' subgroup (1.1[95% CI = 0.2 to 2.0] versus 8.6 [95% CI = 8.4 to 8.7]).

**Table 2.** Comparison of most frequently prescribed CAPS medication subgroups, non-ID group versus ID group. Top 10 CAPS medication subgroups ranked by frequency

Medication subgroup	Medication subgroup rank for ID group (age–sex standardised)	Medication subgroup rank for non-ID group
Antipsychotics^a^	1	36
Anticonvulsants^a^	2	37
Broad spectrum penicillins	3	2
Immunisation	4	14
Antidepressants	5	4
Simple analgesics	6	10
Penicillins or cephalosporins	7	3
Antiulcerants	8	9
Sex or anabolic hormones	9	23
Other antibiotics^a^	10	8
Antihypertensives^a^	18	1
Other cardiovascular system^a^	13	5
NSAIDs^a^	38	6
Narcotic analgesics^a^	20	7

^a^Non-overlapping 95% confidence intervals, comparing age–sex standardised ID with non-ID group.

ID = intellectual disability. NSAID = non-steroidal anti-inflammatory drug.

#### Psychoactive medication subgroup recommendations

Anticonvulsants and antipsychotics were recommended significantly more often at ID group encounters compared with non-ID group encounters (Table 3). There was no difference in recommendation rates for antidepressant, anti-anxiety, and sedative or hypnotic medication subgroups.

**Table 3.** Comparison between the ID (age–sex standardised) and non-ID groups of selected nervous system medication subgroups: rates per 100 encounters.

Medication subgroup	ID group		ID group (age–sex standardised)	Non-ID group	
	*n*	Per 100 encounters (95% CI)	Per 100 encounters (95% CI)	*n*	Per 100 encounters (95% CI)
Anticonvulsants^a^	54	7.8 (4.7 to 11.0)	6.0 (3.2 to 8.7)	5764	0.6 (0.6 to 0.6)
Antipsychotics^a^	48	7.0 (4.8 to 9.1)	6.1 (3.7 to 8.5)	5837	0.6 (0.6 to 0.6)
Antidepressants	30	4.3 (2.7 to 6.0)	3.5 (1.9 to 5.2)	36 054	3.7 (3.6 to 3.8)
Antianxiety	12	1.7 (0.8 to 2.7)	1.6 (0.6 to 2.6)	18 859	1.9 (1.9 to 2.0)
Sedatives or hypnotics	10	1.4 (0.6 to 2.3)	1.3 (0.4 to 2.3)	16 296	1.7 (1.6 to 1.7)

^a^Non-overlapping 95% confidence intervals, comparing ID group (age–sex standardised) with non-ID group.

CI = confidence intervals. ID = intellectual disability.

#### Analgesic subgroup prescriptions

There was no difference between the ID and non-ID groups in the recommendation rate of simple analgesics, but recommendations of narcotic analgesics at ID group encounters was significantly lower than at non-ID group encounters (1.0 [95% CI = 0.0 to 2.3] versus 3.1 [3.0 to 3.2]).

#### Individual medication recommendations (CAPS ‘generic medication’ level)

Compared with non-ID group encounters, the following medications were recommended significantly more often to patients at ID group encounters: sodium valproate (2.9 [95% CI = 1.0 to 4.8] versus 0.2 [95% CI = 0.2 to 0.2]), and medroxyprogesterone (1.8 [95% CI = 0.8 to 2.8] versus 0.3 [0.3 to 0.3]).

## Discussion

### Summary

Analysis of data from a large national primary care database revealed significant between-group prescribing differences at the CAPS medication group, sub-group, and generic medication level. Narcotic analgesics and cardiovascular system medications such as antihypertensives were recommended at significantly lower rates at ID group encounters. However, nervous system medications, specifically antipsychotics and anticonvulsants, were recommended at higher rates at ID group encounters.

### Strengths and limitations

The strengths of this study include the robust BEACH programme methodology that allows for collection of continuous cross-sectional primary care medication data. This is the only Australian data set that allows comparison of national GP medication recommendations at ID and non-ID encounters. However, there are a number of limitations to consider when interpreting study results. Firstly, caution is urged when interpreting results, in view of wide confidence intervals. Secondly, it is likely that ID encounters are under-represented in this data set. For example, at encounters where the person with ID was unable to provide consent and did not have a carer present, the GP would not have included the encounter record as per the BEACH ethics protocol. Some genetic syndromes that have variable association with ID, for example tuberous sclerosis, were also excluded from the coding structure so as to avoid inadvertently including people in the ID group who did not have ID. Thirdly, presence or absence of ID was not collected as part of the patient demographic data in the BEACH programme. While all prescriptions have been allocated to the ID-group where ID was indicated as an RFE or a problem managed, it is possible that prescriptions for some people with ID may have been incorrectly allocated to the non-ID group if ID was not an RFE or a problem managed.

### Comparison with previous literature

Antipsychotics and anticonvulsants were the most frequently recommended CAPS medication subgroups in ID group encounters, but only ranked 36th and 37th respectively in non-ID group encounters. Higher antipsychotic and anticonvulsant recommendations at ID group encounters in this study are consistent with previous literature.^15^ Given the elevated prevalence of mental illness^16^ and epilepsy^17^ in people with ID, the finding that some medications used to treat these conditions are prescribed more frequently at ID group encounters is not unexpected. Previous analysis from this data set, for example, identified that epilepsy was the third most common problem managed at ID group encounters.^8^ However, the significant over-representation of antipsychotic recommendations at ID group encounters may also relate to their use for behavioural control rather than for clearly defined mental illness. Despite limited evidence of efficacy^18,19^ numerous studies have demonstrated high rates of antipsychotic use in people with ID in the absence of a defined mental illness.^15,20,21^

Recommendations for antidepressants and anxiolytics were no higher at ID than non-ID group encounters. This finding is somewhat surprising given well-documented links between ID and increased vulnerability to mental illness.^22^ A previous analysis of the BEACH data set found depression was less likely to be recorded as a problem managed at ID group encounters.^8^ It is possible that diagnostic overshadowing prevented recognition and subsequent treatment of certain mental illnesses in this sample of people with ID. Disorders that are less likely to present with severe disturbances in thoughts or behaviour, such as depression and anxiety, may be particularly challenging to diagnose in primary care.^23^ It is important to note, however, that while a number of studies have indicated elevated rates of depression and/or anxiety in the ID population,^22,24,25^ the exact point prevalence of these conditions is not known.^26^ Diagnostic challenges relating to the detection of depression and anxiety in people with ID, particularly those on the severe or profound spectrum, and inconsistent methodologies applied in past epidemiological studies^26^ mean further clarification of these results is needed.

The lower narcotic analgesia recommendation rates at ID group encounters in this study were another surprising finding, given that a diagnosis of ID is associated with multiple co-morbidities that make the experience of acute and chronic pain more likely than in the general population.^27,28^ As noted previously, this finding should be interpreted with caution due to the relatively wide confidence intervals, although it is consistent with other literature^29,30,31^ that raises questions about the adequacy of pharmacological pain management in people with ID. Clinicians face a number of challenges when assessing pain and analgesic treatment response in people with ID. Many patients with ID lack the ability to self-report the location and/or extent of pain,^32^ or may express pain in idiosyncratic ways.^33^ ID-specific pain management education and access to appropriate non-verbal modified pain scales^34^ may help GPs to identify and adequately treat pain in this population.

The comparatively low rate of cardiovascular medication recommendations, particularly antihypertensives, at ID group encounters is surprising given elevated cardiovascular risk factors associated with a diagnosis of ID.^35^ People with ID are prescribed antipsychotics (many of which have a high cardiometabolic liability) at greater rates, and beginning at an earlier age, than the general population.^36^ They are also less likely to be physically active,^37^ more likely to be overweight,^38^ and more likely to experience genetic syndromes associated with elevated baseline risk.^35^

This study's finding of lower antihypertensive recommendations at ID group encounters is consistent with a number of earlier studies reporting under-detection and under-treatment of chronic cardiovascular health conditions in people with ID.^39^ For example, nearly double the rate of missed metabolic syndrome diagnoses were reported in people with ID compared to the general population in the Dutch Healthy Ageing in Intellectual Disability study,^40^ and hypertension was previously undiagnosed in 50% of the ID cases. It is possible that constraints imposed by administrative ID-associated paperwork — along with the complexity of managing multiple presenting problems simultaneously in a general practice setting^8^ — mean that some clinicians may omit routine procedures such as blood pressure measurement to save time. This may, in turn, contribute to the under-management of hypertension in this population. GPs may also have fewer opportunities to assess the cardiometabolic health of their clients since low levels of health literacy among people with ID mean that as a group they are less likely to present spontaneously to their GP for preventative physical health care.^41^

## Implications for research and practice

This analysis does not examine reasons for differences in prescribing patterns at non-ID and ID encounters. Future research exploring the reasons for differences in prescribing patterns between groups is recommended to clarify the meaning of these findings. However, taken together with other research on prescribing in people with ID, these results may indicate the need for GPs to review prescribing in patients with ID, with a focus on:

- correcting potential under-treatment of affective mental disorders in people with ID, including depression and anxiety;
- evaluating potential over-reliance on psychotropic medications for behavioural management (the NHS clinical guideline for medication use in populations with challenging behaviour may be a useful reference point for best practice^20^); and
- examining potential under-treatment of pain and other core health concerns in populations with ID. Lower than expected narcotic analgesic and cardiovascular recommendations at ID group encounters suggest that greater focus could be placed on screening for core health concerns in this population. A number of screening and treatment tools specifically for people with ID may be of use. For information on modified pain scales for non-verbal patients, see Breau *et al;* ^34^ for cardiometabolic screening and treatment guidelines for people with ID, see Trollor *et al.* ^42^

Medication recommendations made by Australian GPs differ for people with ID and for controls. People with ID were recommended antipsychotics and anticonvulsant at higher rates, and had altered patterns of analgesic recommendations and lower rates of antihypertensive recommendations. Further research linking diagnostic and prescribing data would assist in clarifying reasons for prescribing in people with ID. Future active monitoring of medication recommendations in primary care is recommended to inform efforts to address the health status of people with ID. Monitoring may support targeted initiatives^3^ including training, education, toolkits, and medicines reviews, thus improving prescribing practices among primary care providers. Administrative incentives to encourage proper assessment of complex cases^43^ may also encourage proactive and preventative primary healthcare provision to this population.
